# Innovations in technology and service delivery to improve Retinopathy of Prematurity care

**Published:** 2018

**Authors:** Anand Vinekar, Pramod Bhende

**Affiliations:** Programme Director: KIDROP, Professor & HoD: Department of Pediatric Retina Narayana Nethralaya Eye Institute, Bangalore, India.; Director: Shri Bhagwan Mahavir Department of Vitreo Retinal Services, Sankara Nethralaya, Chennai, India.

**Figure:**
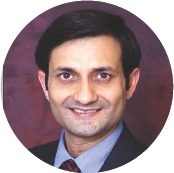
Anand Vinekar

**Figure:**
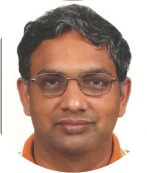
Pramod Bhende

**A novel method of using mobile screening by non-physician imagers can address a key challenge of lack of ROP specialists in the South Asia region.**

**Figure 1A:**
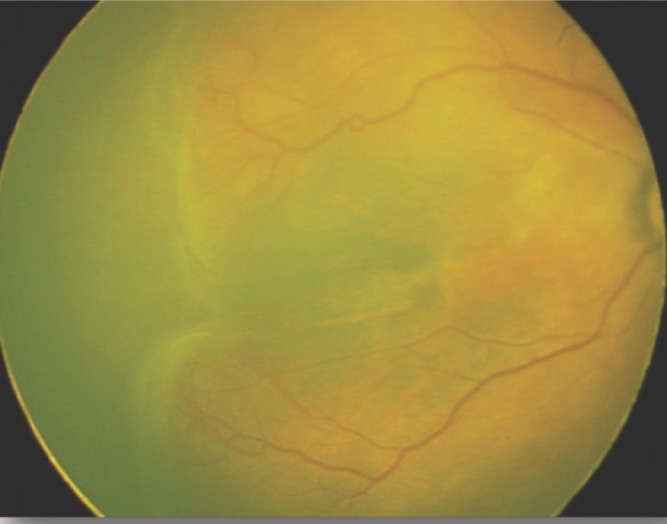
RetCamShuttle (Natus USA) image of Stage 3 ROP in the right eye. The image is rectangular due to the 1800 × 1600 sensor of the camera.

**Figure 1B:**
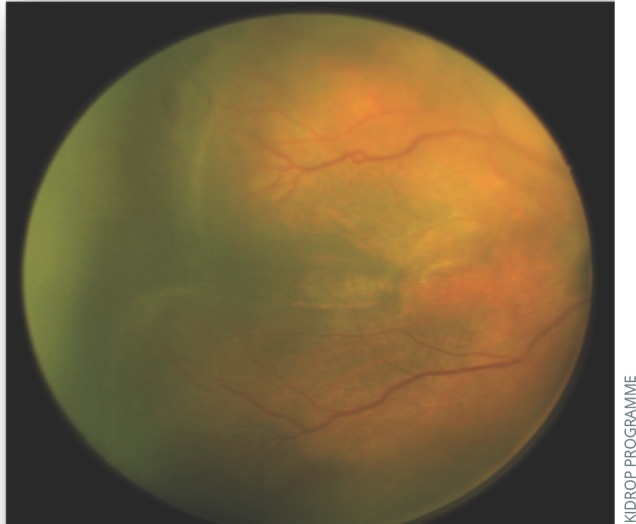
Neo (Forus Health, India) camera of the same eye showing a 2040 × 2040 square image with an additional superior and inferior retinal arc.

Improved neonatal care, enhanced preterm survival and a proliferation of neonatal units in middle-income countries has resulted in a steep rise in the number of infants requiring screening for ROP. Unfortunately a gross lack of specialists has resulted in a large gap in the demand and supply equilibrium. A novel method of ‘task shifting’ using mobile screening units manned by non-physician imagers has tried to address this challenge (pp. 9–10). However, there is an unmet deficit that requires innovations in technology and service delivery that can enhance affordability, accessibility and availability of these services even in remote regions. This article focuses on some of these resources especially in the context of middle-income countries.

## Affordable technology for ROP screening

The infant retinal camera of choice for ROP imaging has been the RetCam (Natus USA, formerly Clarity MSI, USA). The portable version of this camera, i.e. the “Shuttle”, has been successfully used to provide ROP screening even in challenging rural conditions in India. A single unit can travel several thousand kilometres in a single week, screening in several units spread across a geographically defined zone. Despite a successful, impactful and scalable working model, the roadblock for larger replicability has been the cost of the camera which is over USD 125,000 in India.

Recently, an indigenously invented wide-field camera from India for ROP screening, the “3Nethra Neo” has become available for commercial use.^1^ The camera provides a 120-degree field of view, is a contact camera with a single, monolithic, hand-held probe. The innovative liquid lens is integrated into the hand-piece and does not require to be removed after each session. The illumination source is a patented warm LED light which has been tested for safety. The image resolution is 2040 × 2040 (compared to 1800 × 1600 of the RetCam) and results in a square image which provides an extra arc of the superior and inferior retina which would be cropped out in the rectangular image of the RetCam (Figure 1A and 1B).

In a pilot study, the “Neo” was evaluated as a ROP screening tool by comparing it with images from the RetCam. Two masked observers reported the diagnosis and decision in over 128 infants, which gave good sensitivity (ability to identify cases) of 97–99%, and good specificity (ability to identify normal eyes) of 75–81%. The study has subsequently been expanded to include over 1,200 infants and the results are encouraging.

**Figure 2:**
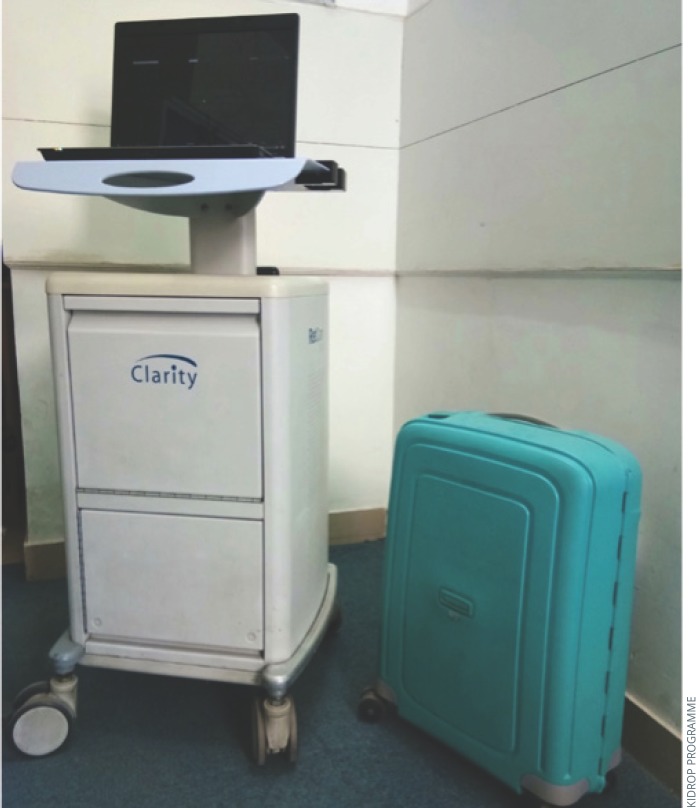
The RetCam Shuttle (left) and the Neo ROP camera (right) adjacent to each other showing their relative sizes.

From the community aspect, the advantages of the Neo are: first, the cost. Currently, in the Indian market, the Neo is one-sixth the cost of the RetCam and with increased demand the price is likely to reduce further. Second, it is smaller and portable, (Figure 2) allowing easier transport (including a two-wheeler) in rural areas and between centres. Third, the Neo has an inbuilt software that allows automatic upload to online servers even in low bandwidth areas, allowing seamless integration into a tele-medicine platform.

With the ubiquitous nature of the smart phone even in rural areas, we are hopeful that in the near future, companies will develop wide-field imaging lens attachments that would allow us to use the mobile phone for ROP screening.

## Online training and e-certification platforms

One of the limitations in scaling up a tele-ROP programme is the lack of a uniform accreditation for imagers. With increasing numbers of “imaging-for-ROP-screening” adopters, it becomes imperative to create trained and accredited imagers who comply with regulatory as well as clinical criteria. Online training modules is one possible solution. For example the 90 working day training of the KIDROP programme (Bangalore, India)^2^ has now become available as an e-learning platform, “WISE-ROP” (Wide-field Imaging for Screening and Education for ROP). Imagers read and undergo self-assessment quizzes at the end of each module. Video sessions and viva voce to correct the technique and practical difficulties are scheduled with an assigned mentor. Graded levels of skill are sought and tested before a certificate is awarded to these new imagers. A report evaluating a tele-ROP programme based on the Center for Disease Control (CDC, Atlanta) guidelines has provided the desired credibility for adopting tele-medicine not only in developing countries, but also in developed nations like Australia.

## Software innovations and artificial intelligence

**Figure 3:**
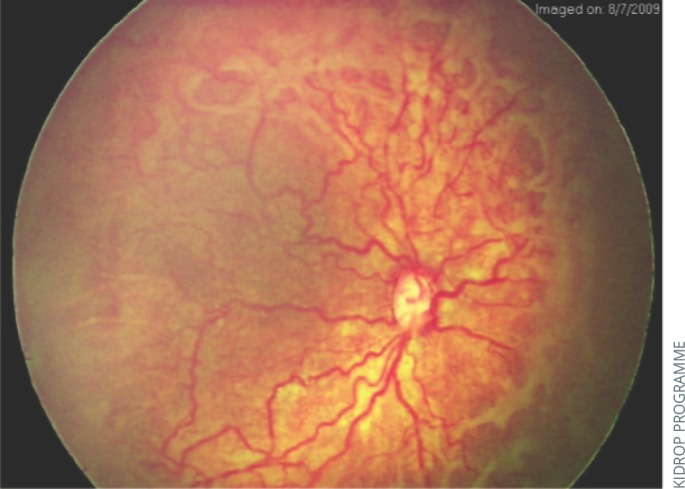
Image processing of a RetCam image using RetiView software that highlights the edge of the disease, the severity of the plus and the elevation of the traction in a case of aggressive posterior ROP (APROP).

**Figure 4:**
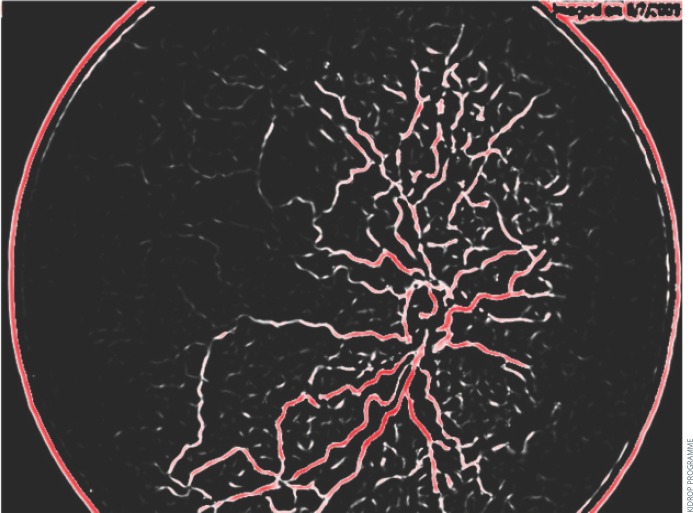
A non-invasive vascular map using the RetiView algorithm which helps the clinician detect smaller capillaries anterior to the clinically visible posterior border of vascularization on the fundus image.

Once retinal images are uploaded on to the server, in the current scenario, experts have to review most of them before either providing or confirming the diagnosis. Given the limited number of experts, this could become an important roadblock as an increasing number of centres switch to image-based screening. Innovations in automated software algorithms are becoming refined to bridge this gap. Disease severity detection, enhancing clinical features of ‘poor quality’ images (Figure 3) and image processing tools to enhance vascular details non-invasively (Figure 4) are now getting integrated into the imaging platform.^3^ With artificial intelligence algorithms already showing promise in other ophthalmic diseases, codes to predict, prognosticate and influence follow-up and management in ROP are in the pipeline.

## Novel service delivery methods

New models of health delivery participation between ROP skilled private institutes or individuals and the government under a ‘public-private-partnership’ or PPP scheme should be encouraged. This has the advantage of state funding combined with private expertise which can be integrated into the country's public health system. Government support has played a major role in promoting ROP tele-health in Mexico, New Zealand and Brazil.

Gilbert et al^4^ suggested another paradigm shift in ROP screening which involves replacing the ophthalmologist-led model with a pediatrician/neonatologist-led model where there is a shortage of ophthalmologists. Neonatologists would monitor their trained nursing staff using a portable low-cost camera to image the babies and an ROP specialist would give an opinion remotely and visit the centre for treatment alone.

Integrating ROP screening with universal eye screening, using imaging is being practiced in parts of India and China. This model serves a larger population of infants and thus, reduces screening costs.

## Creating stronger surveillance

ROP has gained medical and legal significance in recent years. After a landmark judgment in 2015 by the Supreme Court of India effectively making ROP screening mandatory, surveillance of ROP screeners and treatment providers has become even more important. The National Task force of ROP in India is now an apex body that regulates and promotes such activity. The Indian Retinopathy of Prematurity (iROP) Society,^5^ which comprises of ROP skilled ophthalmologists have united to collaborate on best care practices that are ethical, evidence-based and address local needs.

## Conclusion

Innovations in ROP management are constantly improving the accessibility, affordability and availability of care for these tiny and precious babies.
